# Supplementary material for: Impact of TMB/PD-L1 expression and pneumonitis on chemoradiation and durvalumab response in stage III NSCLC
Source: Nat Commun. 2023 Jul 15;14:4238. doi: 10.1038/s41467-023-39874-8 (PMC10349822; doi:10.1038/s41467-023-39874-8)
Supplement: Supplementary file 1 — Supplementary Information [file 41467_2023_39874_MOESM1_ESM.pdf]

Supplementary Information

Impact of TMB, PD-L1 expression, and pneumonitis on outcomes to chemoradiation and durvalumab in stage III NSCLC

Alessi, et al

Contents

Supplementary Figures. 1-17

Supplementary Tables. 1-6

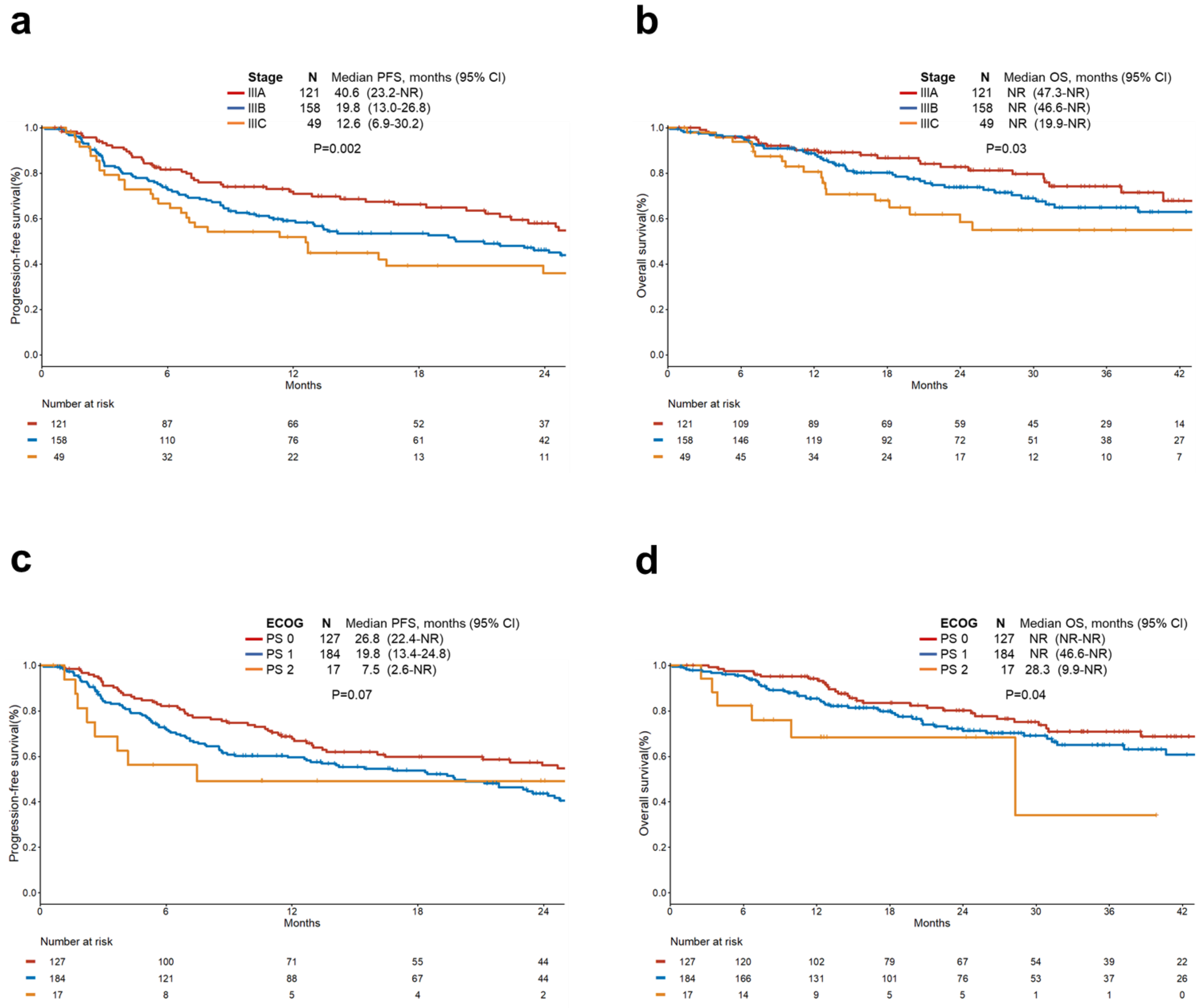

Supplementary Figure 1**. Impact of clinicopathologic factors on outcomes to chemoradiation and durvalumab.** Progression-free (PFS), and overall survival (OS) according to **(a-b)** disease stage and **(c-d)** ECOG PS in patients treated with chemoradiation and durvalumab. P-values were calculated using log-rank analysis. NR, not reached. Source data are provided as a Source Data file.

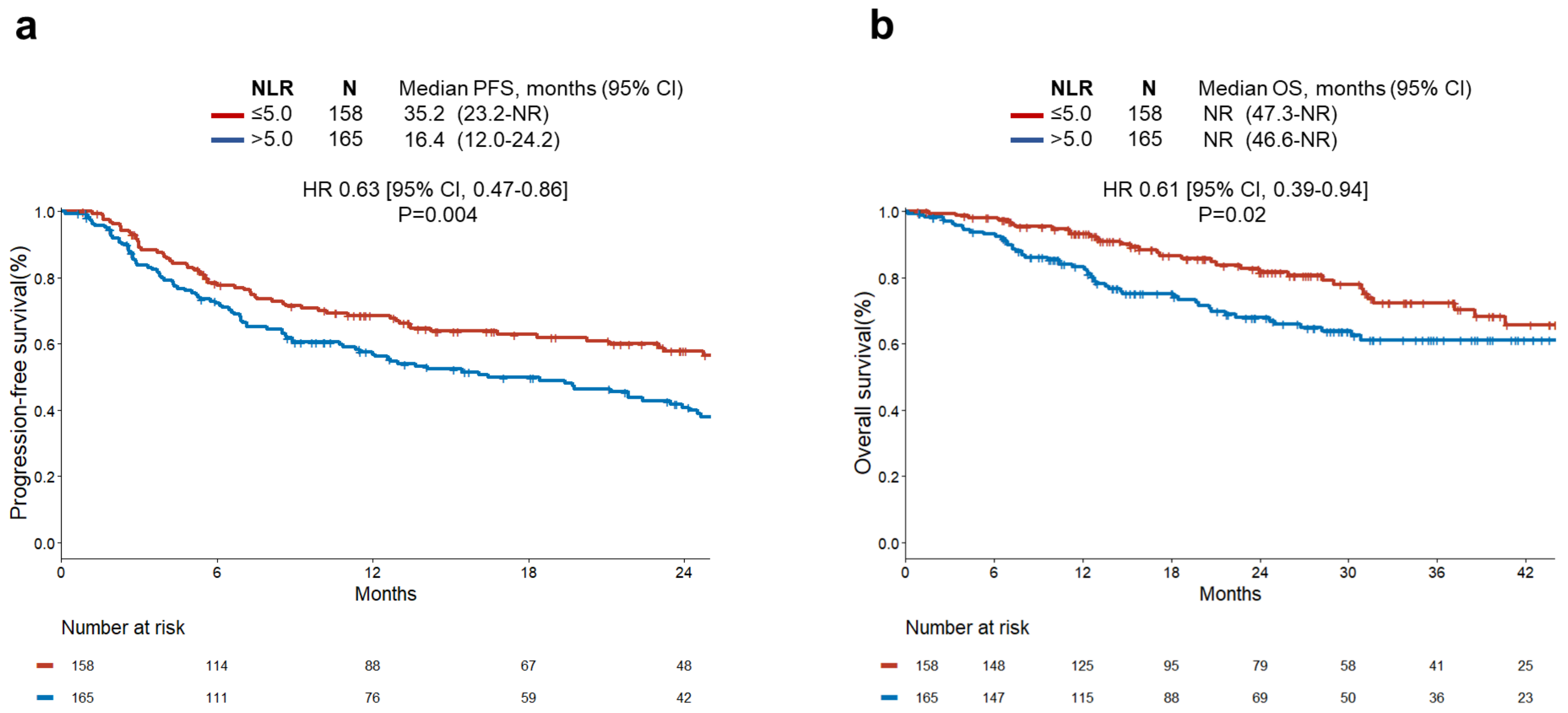

Supplementary Figure 2**. Outcomes to chemoradiation and durvalumab according to NLR level. (a)** Progression-free (PFS) and **(b)** overall survival (OS) according to neutrophil-lymphocyte ratio (NLR) prior to durvalumab initiation. Hazard ratio (HR) and P-values were calculated using unadjusted Cox proportional hazard regression models. NR, not reached. Source data are provided as a Source Data file.

a

Pairwise comparisons between PD-L1 expression groups in terms of local-regional control at 24-months.

PD-L1 TPS groups	HR	CI (95%)	P-value
≥90% vs 50-89%	0.50	0.14-1.85	0.30
≥90% vs 1-49%	0.32	0.09-1.10	0.07
≥90% vs <1%	0.26	0.08-0.86	0.03
50-89% vs 1-49%	0.62	0.27-1.41	0.30
50-89% vs <1%	0.51	0.24-1.11	0.09
1-49% vs <1%	0.83	0.43-1.58	0.57

b

Pairwise comparisons between PD-L1 expression groups in terms of distant control at 24-months.

PD-L1 TPS groups	HR	CI (95%)	P-value
≥90% vs 50-89%	0.66	0.29-1.54	0.30
≥90% vs 1-49%	0.45	0.21-0.97	0.04
≥90% vs <1%	0.37	0.17-0.79	0.01
50-89% vs 1-49%	0.65	0.36-1.18	0.20
50-89% vs <1%	0.62	0.36-1.09	0.10
1-49% vs <1%	1.12	0.71-1.76	0.62

Supplementary Figure 3**. Local-regional and distant control rates according to PD-L1 expression groups.** Pairwise comparisons between PD-L1 expression groups in terms of **(a)** local-regional and **(b)** distant control at 24-months. Hazard ratio (HR) and P-values were calculated using unadjusted Cox proportional hazard regression models. Source data are provided as a Source Data file.

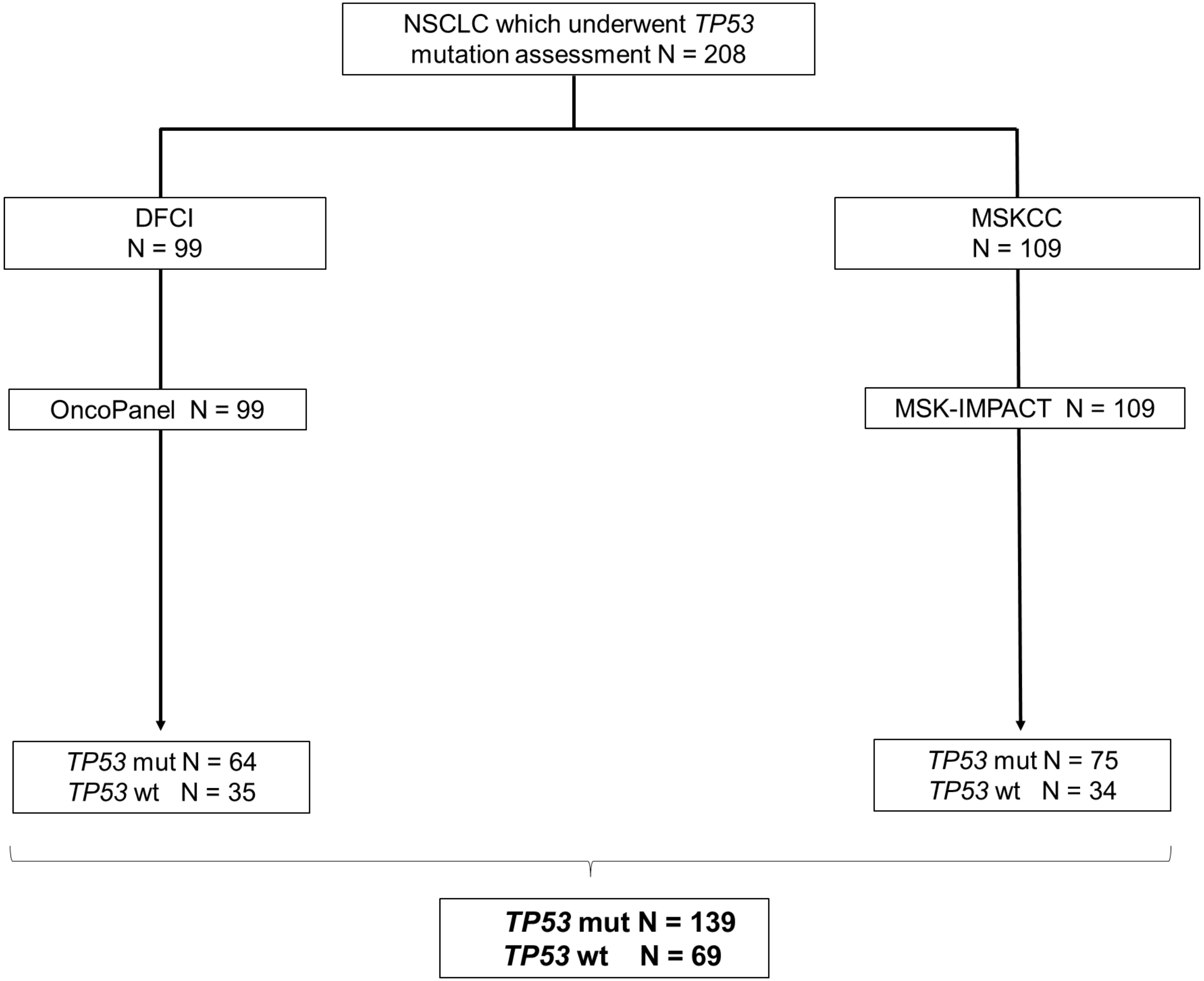

Supplementary Figure 4. **Consort diagram** showing the cohorts of patients in whom *TP53* mutation status was determined. DFCI, Dana-Farber Cancer Institute; MSKCC, Memorial Sloan Kettering Cancer Center.

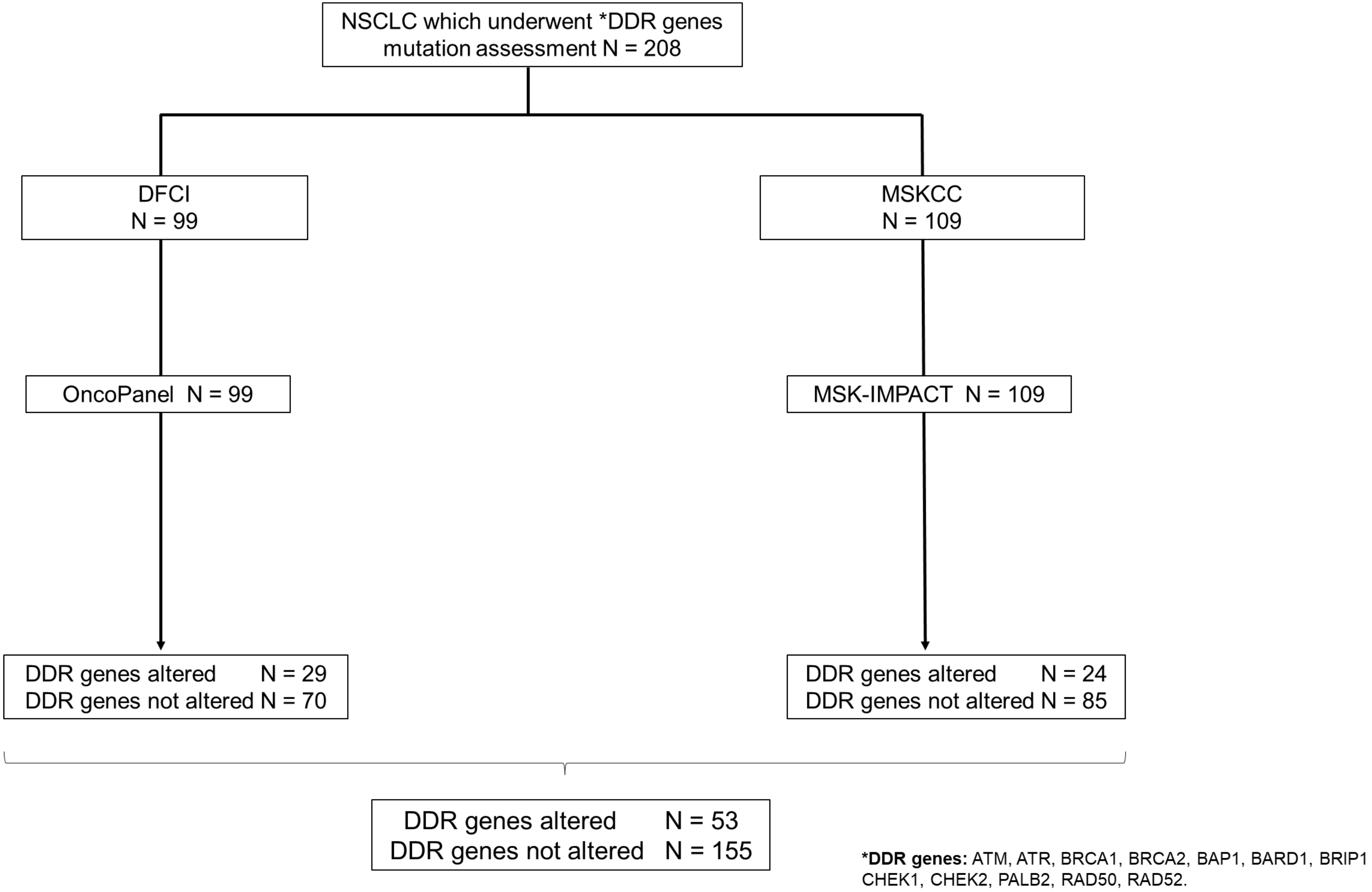

Supplementary Figure 5. **Consort diagram** showing the cohorts of patients in whom DNA-damage repair (DDR) mutation status was determined. DFCI, Dana-Farber Cancer Institute; MSKCC, Memorial Sloan Kettering Cancer Center.

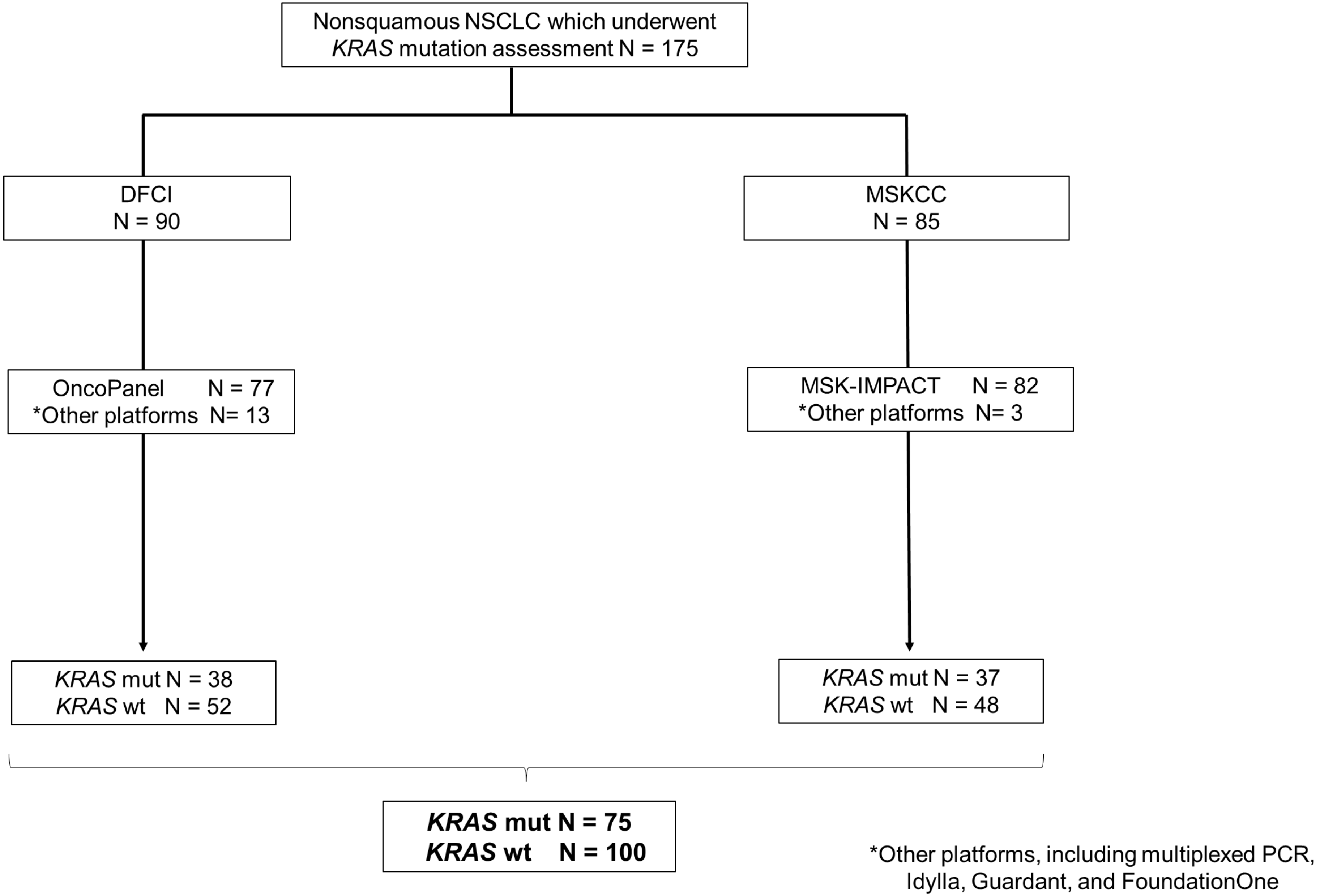

Supplementary Figure 6**. Consort diagram** showing the cohorts of patients in whom *KRAS* mutation status was determined. DFCI, Dana-Farber Cancer Institute; MSKCC, Memorial Sloan Kettering Cancer Center.

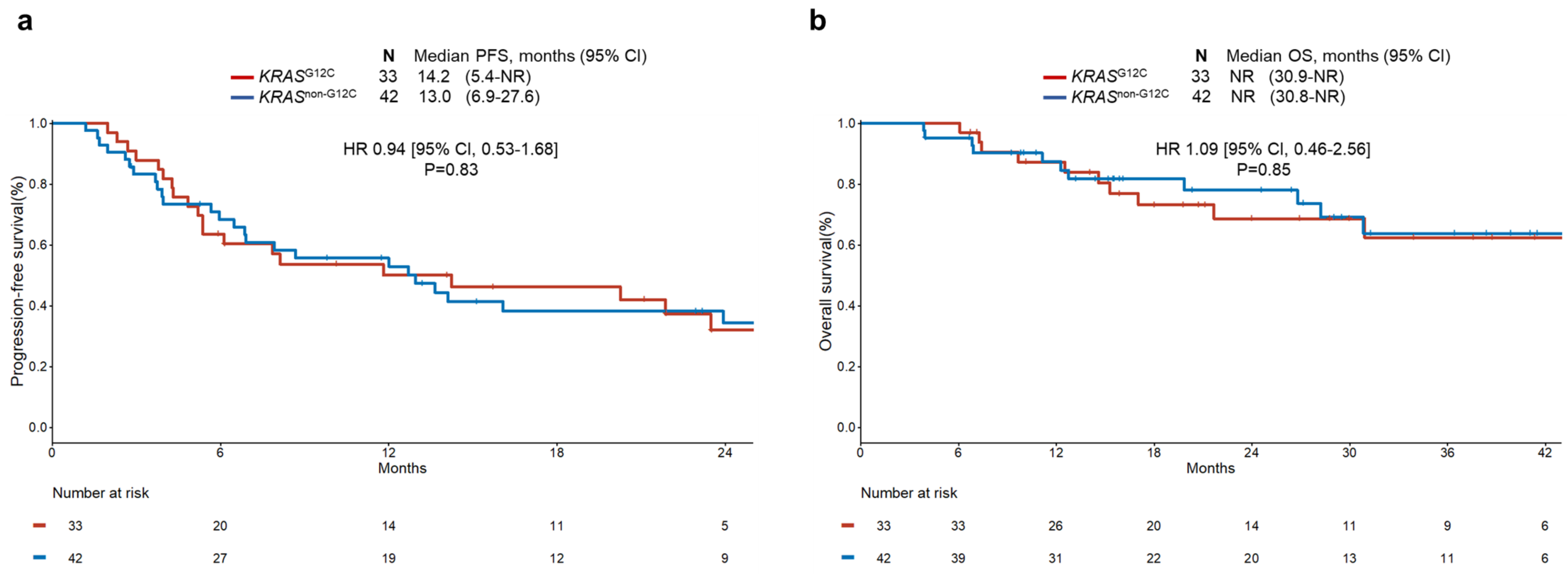

Supplementary Figure 7. **Outcomes to chemoradiation and durvalumab in *KRAS*^{G12C} vs *KRAS*$^{non\text{-}G12C}$. (a)** Progression-free (PFS) and **(b)** overall survival (OS) to durvalumab consolidation by *KRAS* allele subtypes (*KRAS*^{G12C} vs *KRAS*$^{non\text{-}G12C}$). Data are presented as the hazard ratio (HR) with error bars showing 95% confidence interval (CI). HR and P-values were calculated using unadjusted Cox proportional hazard regression models. Source data are provided as a Source Data file.

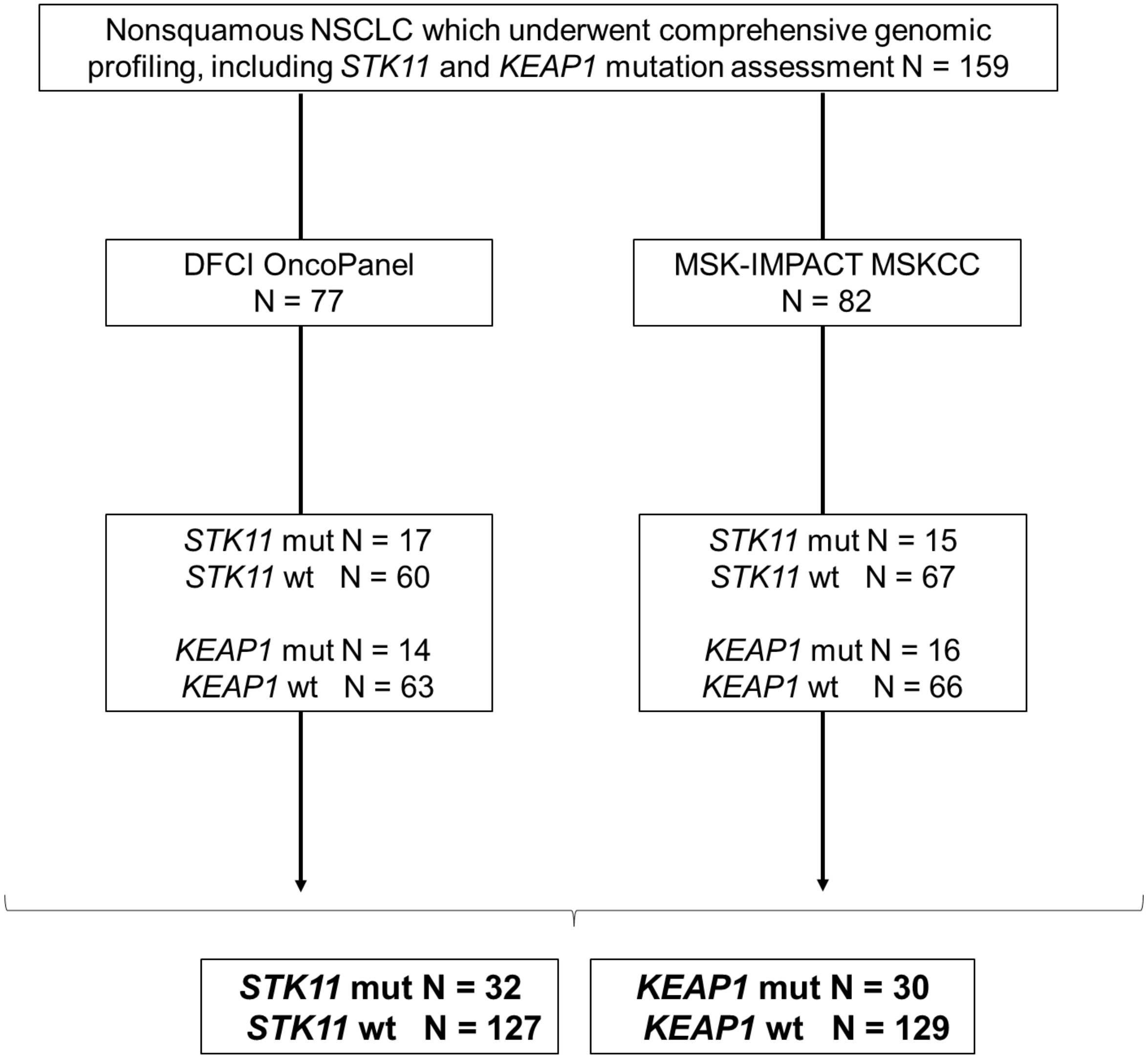

Supplementary Figure 8. **Consort diagram** showing the cohorts of patients in whom *STK11 and KEAP1* mutation status were determined. DFCI, Dana-Farber Cancer Institute; MSKCC, Memorial Sloan Kettering Cancer Center.

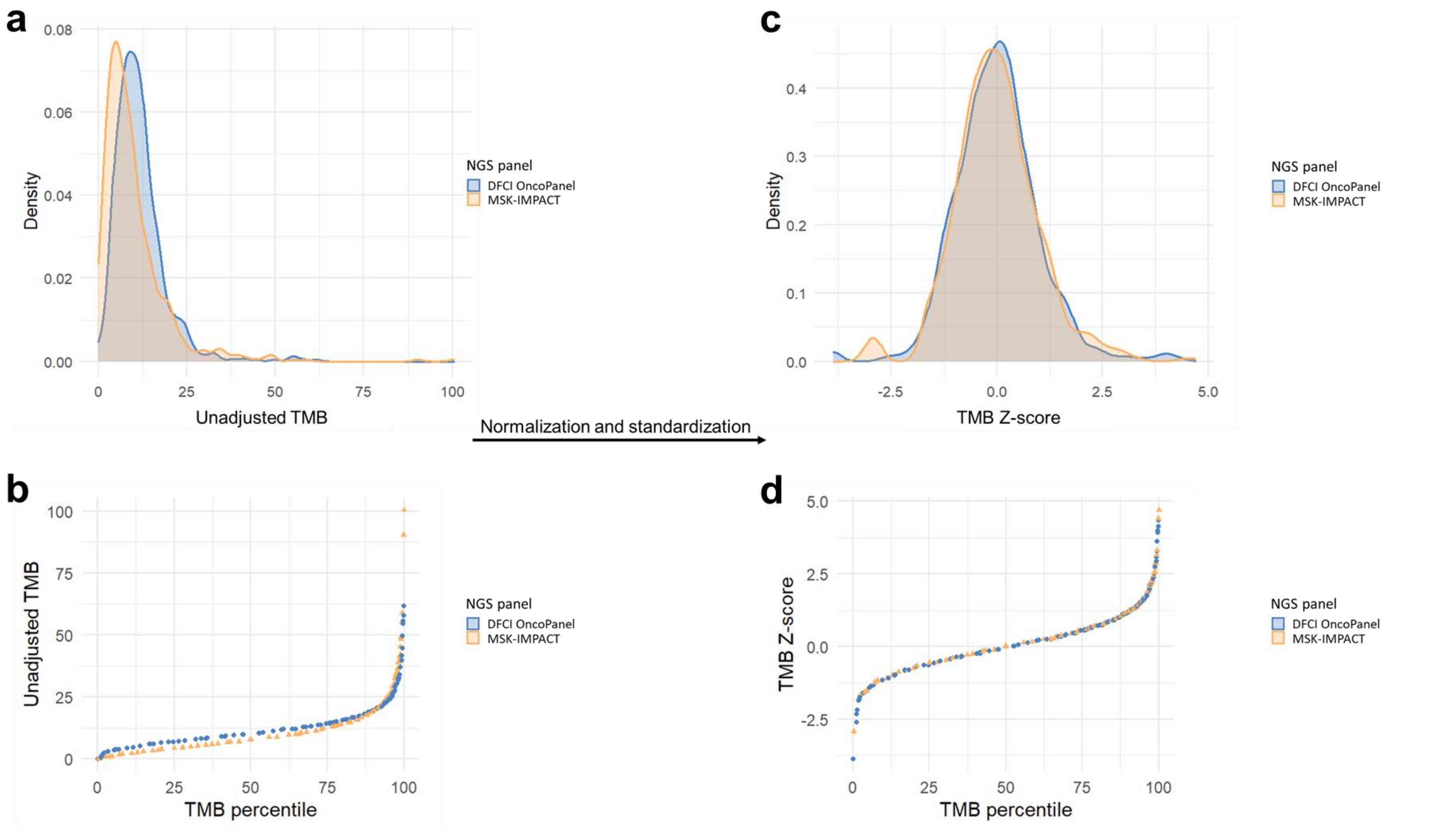

Supplementary Figure 9. **Normalization and standardization of TMB distributions bring the Next Generation Sequencing (DFCI-OncoPanel and MSK-IMPACT) distributions into alignment.** The left side shows the Kernel density plot of unadjusted TMB values in each cohort **(a-b)**, and the right side shows the transformed density plot of TMB Z-scores that demonstrate high overlap **(c-d)**.

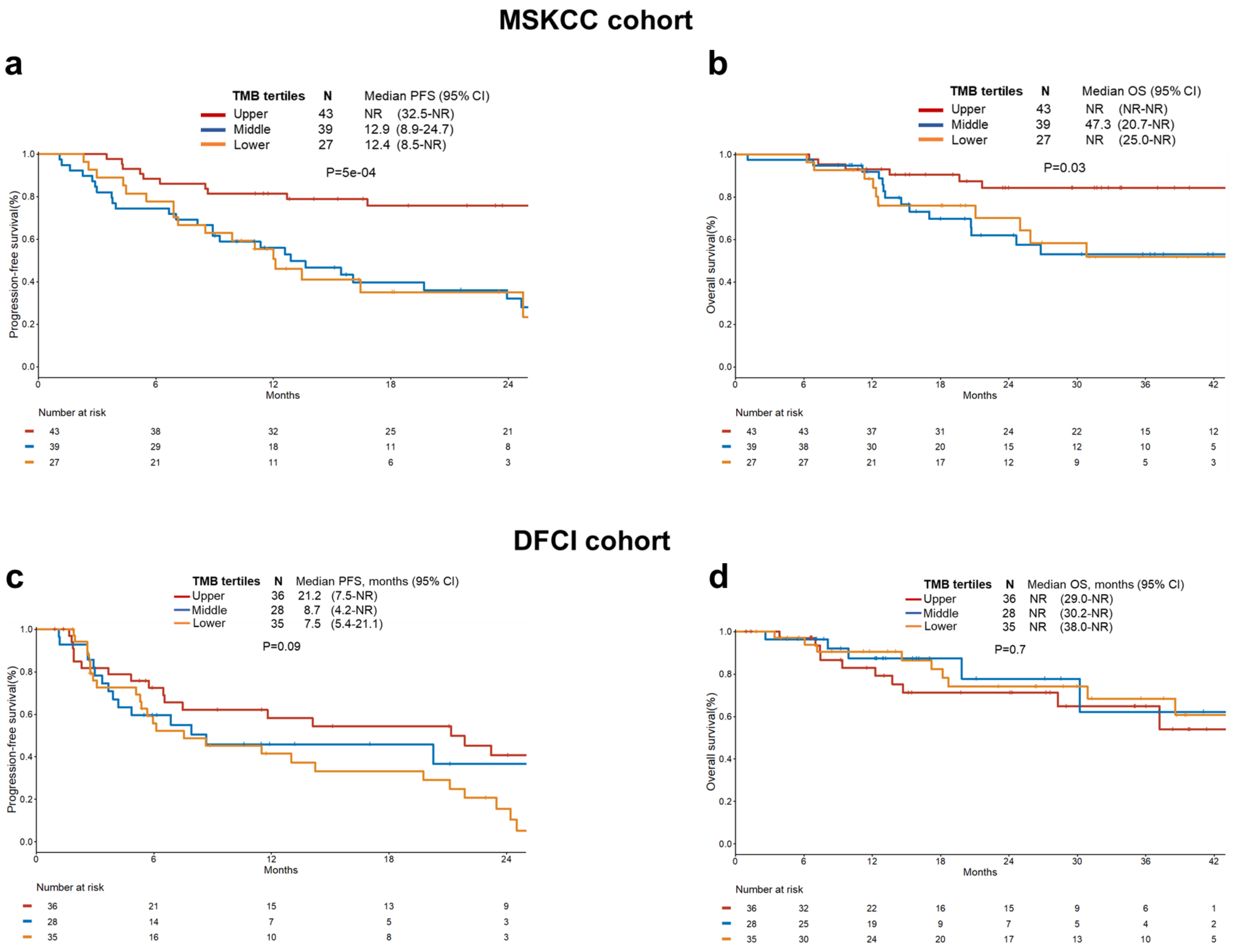

Supplementary Figure 10. **Outcomes to chemoradiation and durvalumab according to TMB tertiles.** Progression-free (PFS) and overall survival (OS) by TMB tertiles in MSKCC cohort **(a-b)** and in the DFCI cohort **(c-d)**. P-values were calculated using log-rank analysis. NR, not reached. Source data are provided as a Source Data file.

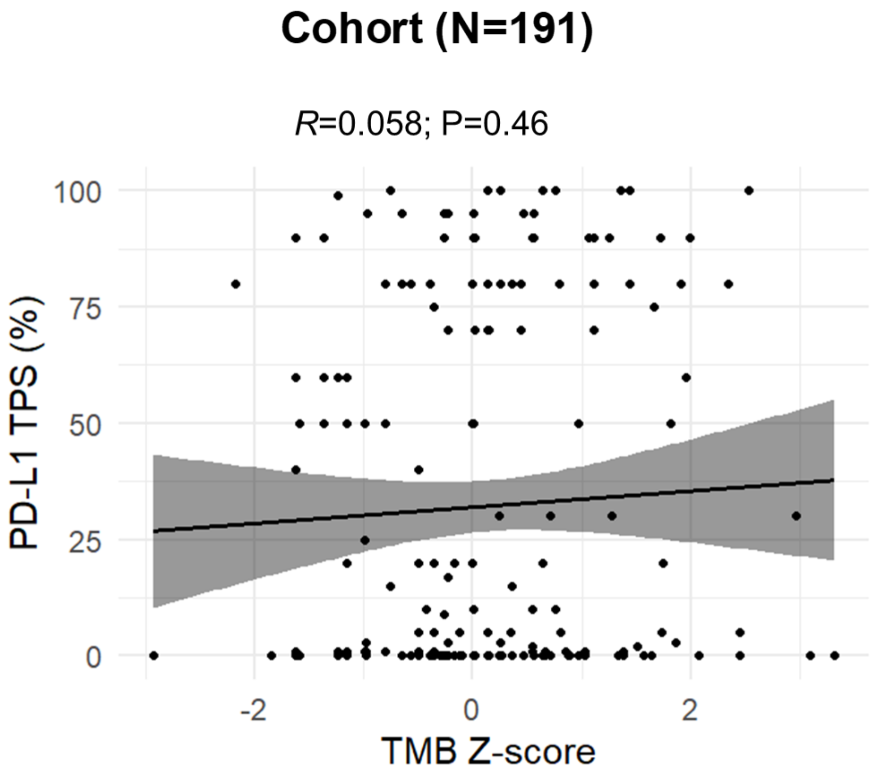

Supplementary Figure 11. **Spearman's rank test.** Spearman's correlation coefficient (*R*) between PD-L1 TPS (%) and TMB Z-score as continuous variable. P-values were calculated using log-rank analysis. Source data are provided as a Source Data file.

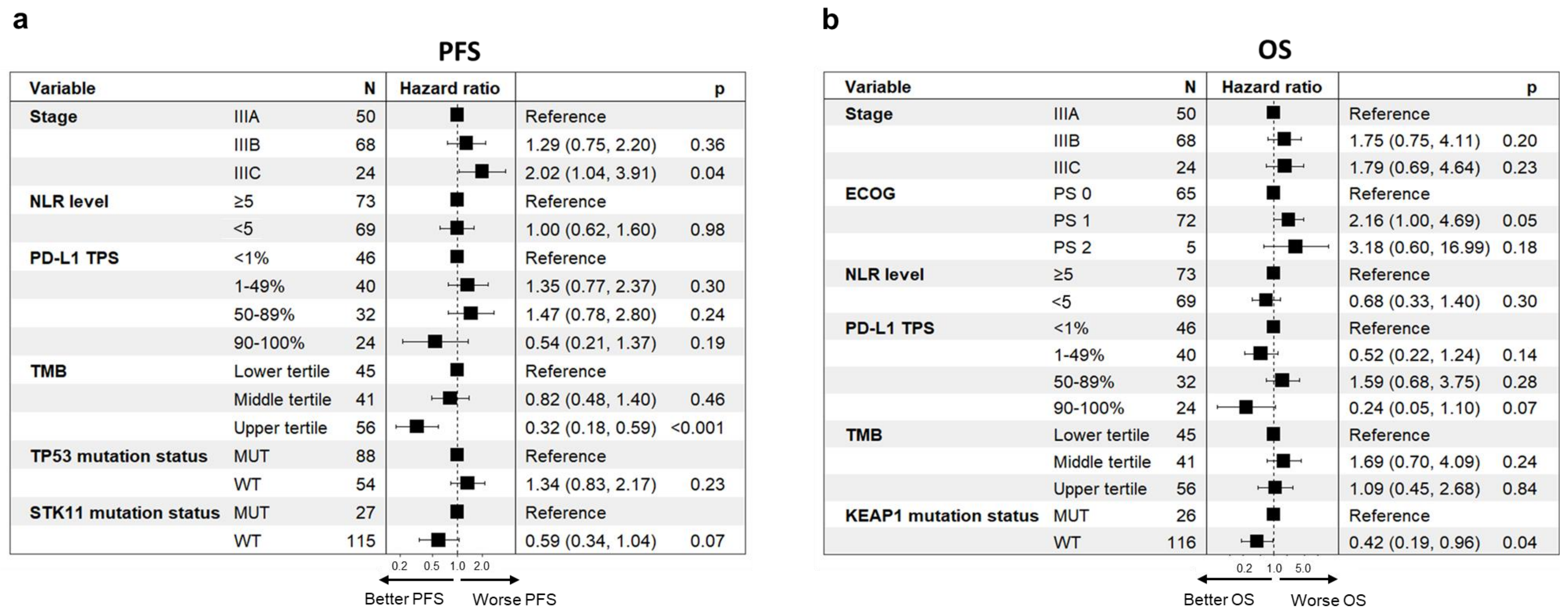

Supplementary Figure 12. **Multivariable Cox regression analysis**. Forest plot for **(a)** progression-free and **(b)** overall survival in multivariable Cox regression analysis in the cohort of patients with stage III nonsquamous NSCLC treated with chemoradiation and durvalumab. Data are presented as the hazard ratio (HR) with error bars showing 95% confidence interval (CI). HR and P-values were calculated using adjusted Cox proportional hazard regression models. Source data are provided as a Source Data file.

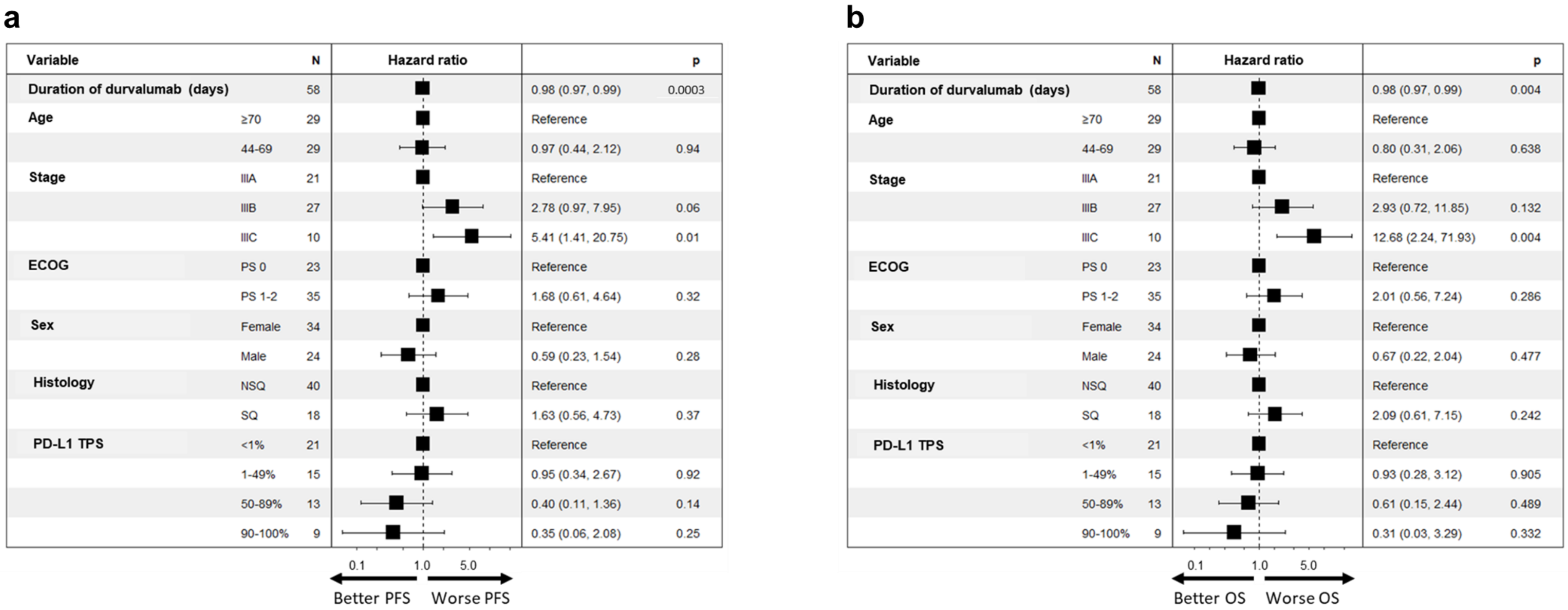

Supplementary Figure 13. **Multivariable Cox regression analysis**. Forest plot for **(a)** progression-free and **(b)** overall survival in multivariable Cox regression analysis including duration of durvalumab treatment prior to discontinuation as a continuous time-dependent variable. Data are presented as the hazard ratio (HR) with error bars showing 95% confidence interval (CI). HR and P-values were calculated using adjusted Cox proportional hazard regression models. Source data are provided as a Source Data file.

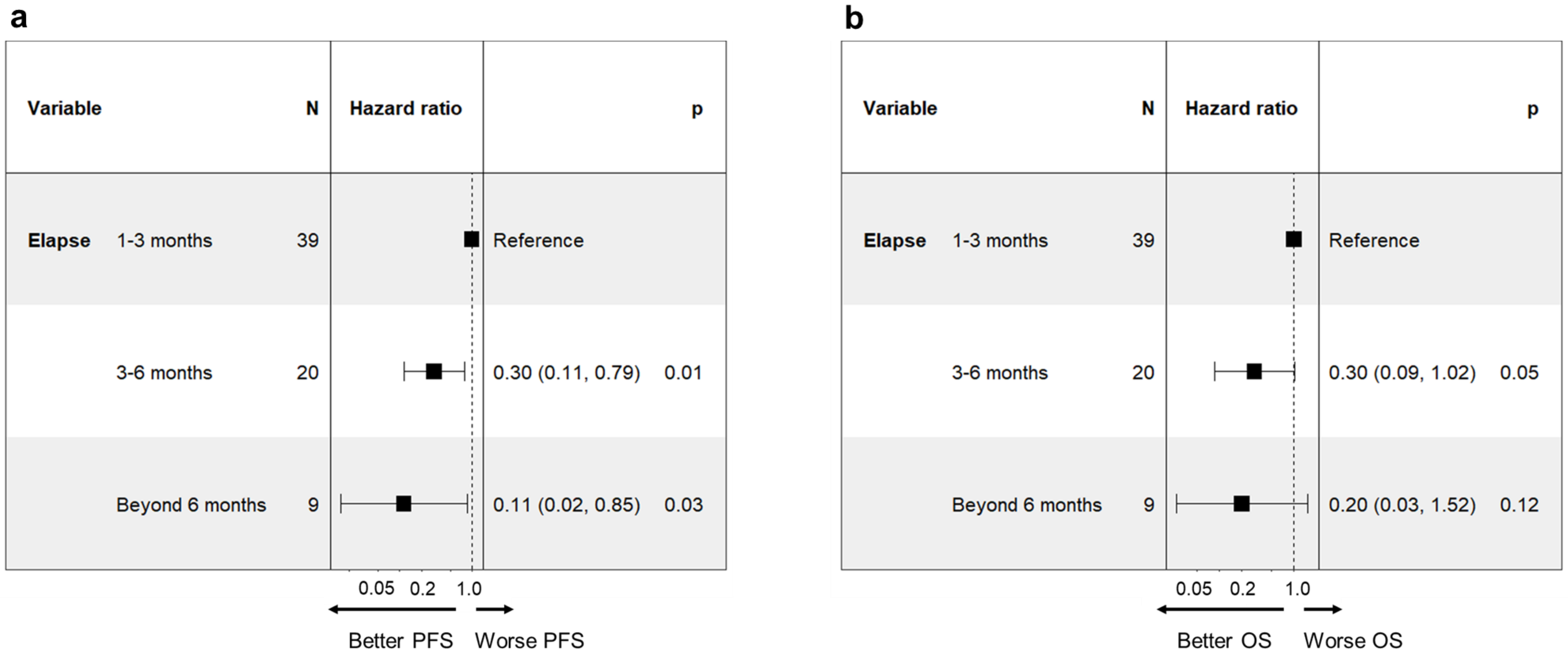

Supplementary Figure 14. **Association of pneumonitis and its timing with disease outcomes.** Forest plot for **(a)** progression-free and **(b)** overall survival in time-dependent Cox regression model of patients who developed pneumonitis including its latency as an ordinal variable (<3 months vs 3-6 months vs >6 months). Hazard ratio (HR) and P-values were calculated using unadjusted Cox proportional hazard regression models. Source data are provided as a Source Data file.

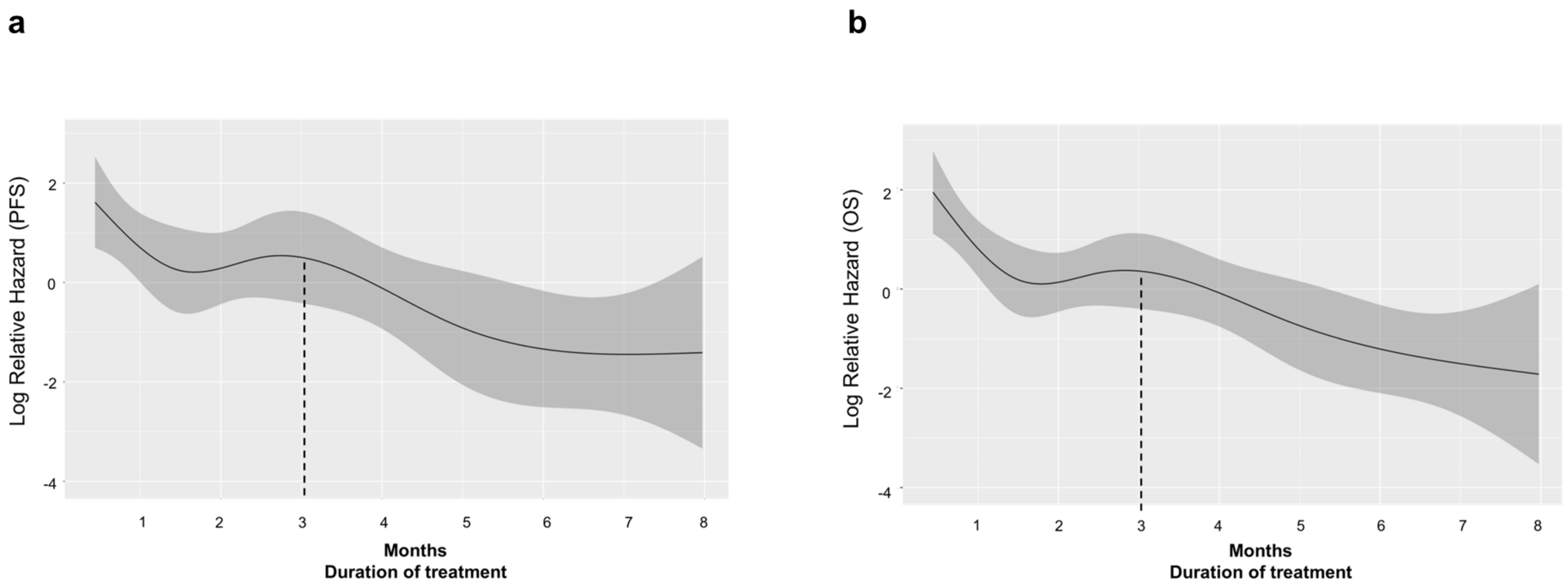

Supplementary Figure 15. **Cubic Spline Regression**. Hazard ratio of duration of durvalumab treatment from **(a)** progression-free survival (PFS) and **(b)** overall survival (OS) in univariable Cox model. Restricted cubic spline was applied to duration of durvalumab treatment with the reference of 3 months (early vs late-onset pneumonitis). 95% confidence intervals are reported under each curve estimates and as shadowed area from the restricted-cubic-spline model. Source data are provided as a Source Data file.

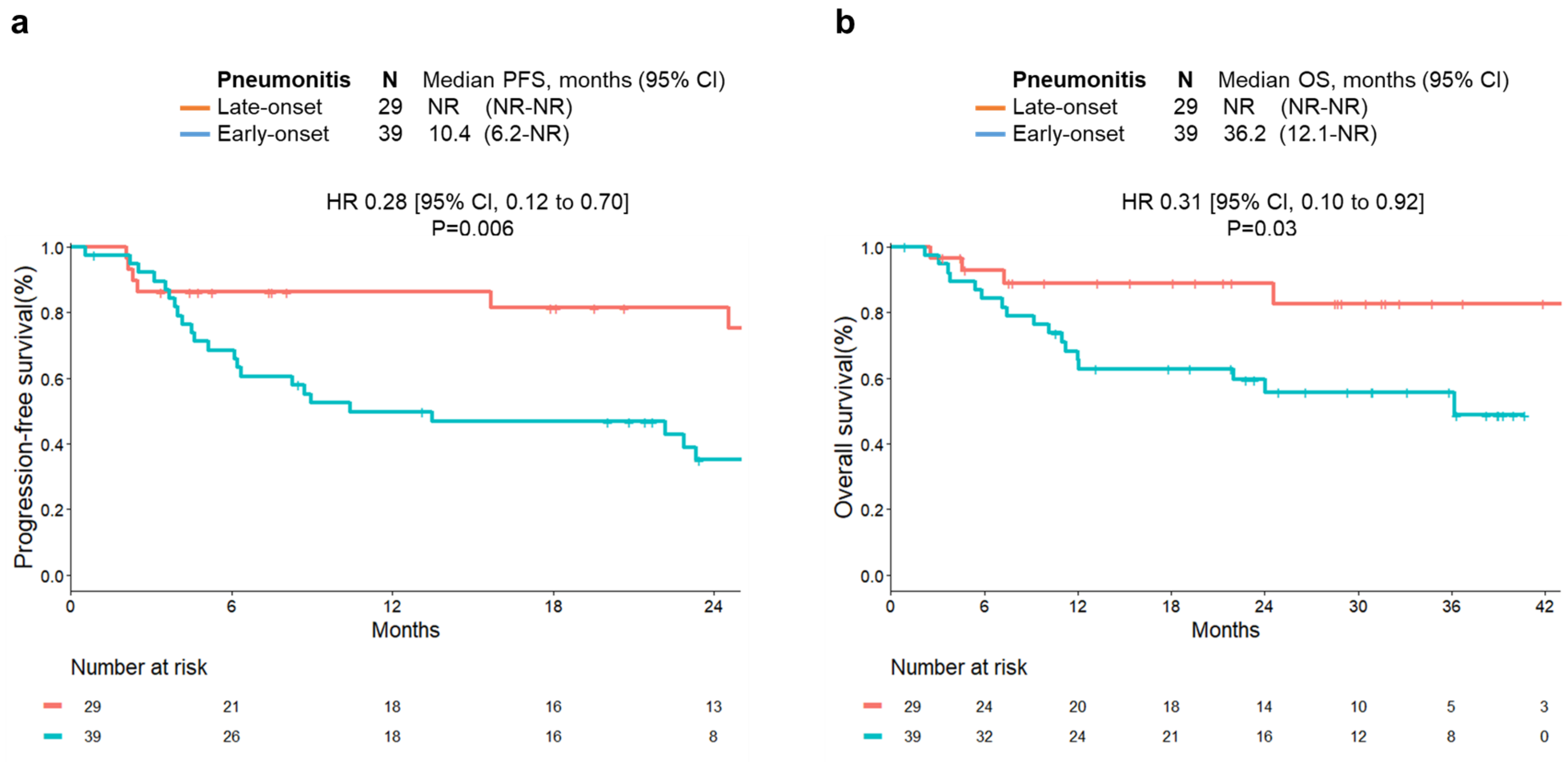

Supplementary Figure 16. **Disease outcomes after development of pneumonitis. (a)** Progression-free (PFS) and **(b)** overall survival (OS) after the development of pneumonitis and discontinuation of durvalumab among patients who experienced early-onset pneumonitis (<3 months) versus late-onset pneumonitis (≥3 months). Data are presented as the hazard ratio (HR) with error bars showing 95% confidence interval. HR and P-values were calculated using unadjusted Cox proportional hazard regression models. NR, not reached. Source data are provided as a Source Data file.

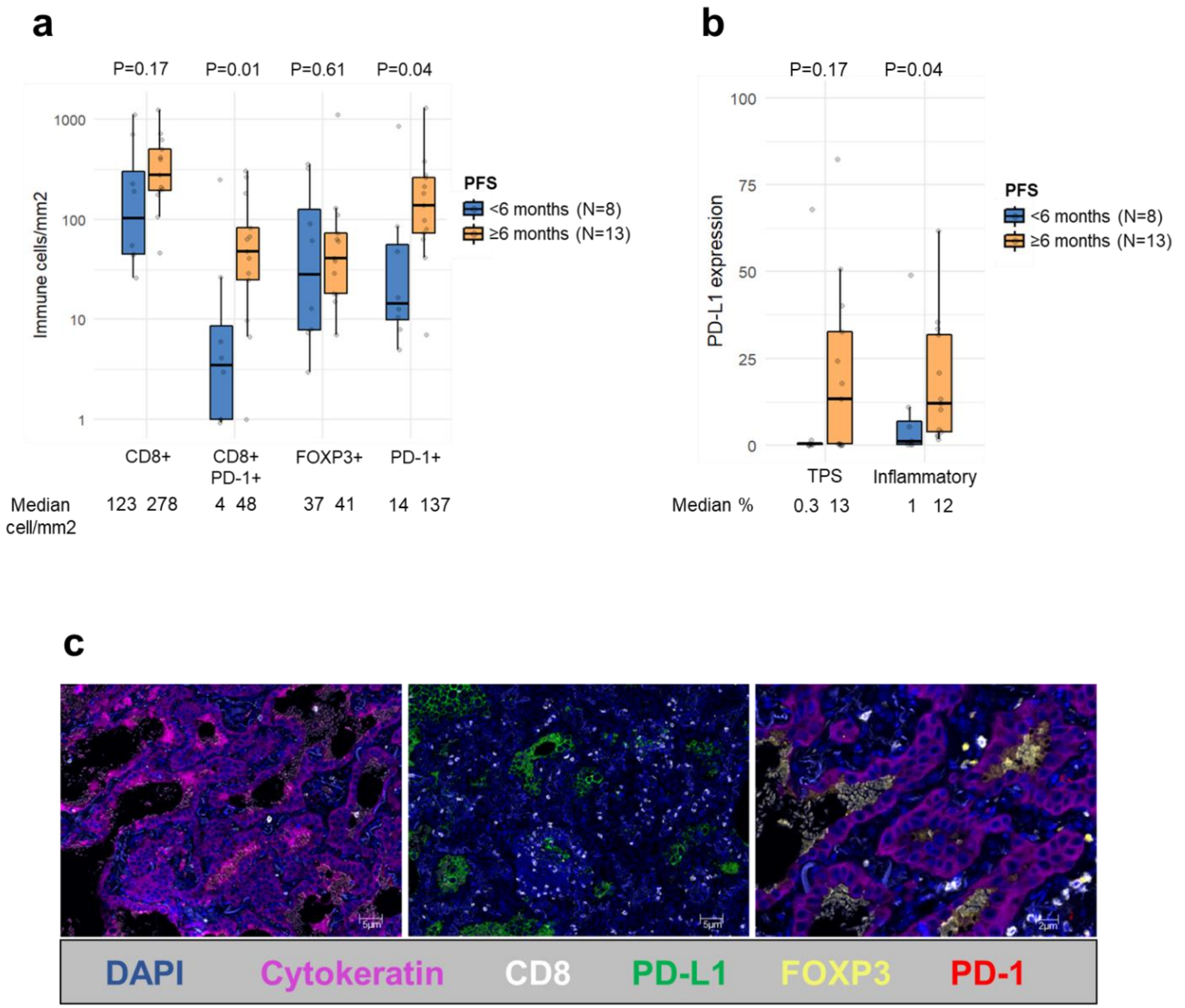

Supplementary Figure 17**. Tumor immunophenotype and disease outcomes. (a)** Median number of tumor-associated immune cells (CD8+, double positive PD-1+ CD8+, FOXP3+, and PD-1+ immune cells) and **(b)** PD-L1 expression on tumor and immune cells in NSCLCs from patients who experienced mPFS ≥ or <6 months as best response to durvalumab. **(c)** Multiplexed immunofluorescence using the ImmunoProfile platform on 3 samples from NSCLCs. P-values are according to Wilcox-rank test for **a** and **b**. Bounds of box plots correspond to interquartile range (IQR, 25-75th percentile). The upper limit of whiskers is the largest value within 1.5 times IQR range above 75th percentile. The lower limit of whiskers is the smallest value within 1.5 times IQR below 25th percentile. Source data are provided as a Source Data file.

Supplementary Table 1. Clinicopathologic and genomic characteristics of 328 patients who received durvalumab after chemoradiotherapy by academic center.

**Clinical Characteristic**	**DFCI N=148 (%)**	**MSKCC N=180 (%)**
**Age,** median (range)	69 (44-86)	67 (45-86)
**Sex**		
Male	65 (43.9)	105 (58.3)
Female	83 (54.1)	75 (41.7)
**ECOG**		
PS 0	35 (23.6)	92 (51.1)
PS 1	96 (64.9)	88 (48.9)
PS 2	17 (11.5)	0
**Smoking status**		
Current/Former	139 (93.9)	174 (96.7)
Never	9 (6.1)	6 (3.3)
**Histology**		
Nonsquamous	105 (70.9)	123 (68.3)
Squamous	43 (29.1)	57 (31.7)
**Oncogene Driver (NSQ)***		
KRAS	38 (42.2)	37 (43.5)
EGFR	3 (3.3)	0 (0.0)
Others	9 (10.0)	9 (10.6)
None identified	40 (44.5)	39 (45.9)
Not assessed	15	38
**TMB (mut/Mb),** median (range)±	9.9 (1.5-42.5)	8.8 (0-68.5)
**PD-L1 TPS**		
≥90%	21 (16.7)	19 (12.8)
50-89%	32 (25.4)	29 (19.6)
1-49%	34 (27.0)	41 (27.7)
<1%	39 (31.0)	59 (39.9)
Not assessed	22	32
**Stage (AJCC 8th Edition)**		
IIIA	73 (49.3)	48 (26.7)
IIIB	56 (37.8)	102 (56.7)
IIIC	19 (12.8)	30 (16.7)
**Radiation dose**		
54-58.4 Gy	8 (5.4)	4 (2.2)
60 Gy	109 (73.7)	156 (86.7)
62-70 Gy	31 (20.9)	20 (11.1)
**Chemotherapy regimen**		
Carboplatin + Paclitaxel	70 (47.3)	81 (45.0)
Carboplatin + Pemetrexed	27 (18.2)	48 (26.7)
Cisplatin + Pemetrexed	37 (25.0)	35 (19.4)
Cisplatin + Etoposide	14 (9.5)	16 (8.9)

*NSQ: nonsquamous; 175 cases with comprehensive genomic profiling.
Other driver mutations: ALK, BRAF, MET, and HER2/
±TMB assessed by DFCI-OncoPanel (N=99) and MSK-IMPACT (N=109).

Supplementary Table 2. Clinicopathologic characteristics of patients who discontinued durvalumab due to pneumonitis and from patients who did not experience pneumonitis.

Clinical Characteristic	Discontinued N=68 (%)	Not discontinued N=260 (%)	P-value
**Age**			
≥70y	35 (51.5)	118 (45.4)	0.41
<70y	33 (48.5)	142 (54.6)	
**Sex**			
Male	28 (41.2)	142 (54.6)	0.06
Female	40 (58.8)	118 (45.4)	
**T Stage**			
0	10 (14.7)	38 (14.6)	
1	14 (20.6)	49 (18.8)	
2	9 (13.2)	46 (17.7)	0.69
3	18 (26.5)	51 (19.6)	
4	17 (25.0)	76 (29.2)	
**N Stage**			
0	5 (7.4)	8 (3.1)	
1	3 (4.4)	14 (5.3)	0.39
2	37 (54.4)	157 (60.4)	
3	23 (33.8)	81 (31.2)	
**Stage (AJCC 8th Edition)**			
IIIA	26 (38.2)	95 (36.5)	
IIIB	31 (45.6)	127 (48.9)	0.88
IIIC	11 (16.2)	38 (14.6)	
**Radiation dose**			
54-58.4 Gy	0	12 (4.6)	
60 Gy	57 (91.6)	208 (80.0)	0.20
62-70 Gy	11 (8.4)	40 (15.4)	
**RT PTV**,** median (range)	535 (92-1370)	482 (87-1450)	0.56
**Chemotherapy regimen**			
Carboplatin + Paclitaxel	32 (47.0)	119 (45.8)	
Carboplatin + Pemetrexed	15 (22.1)	60 (23.1)	0.77
Cisplatin + Pemetrexed	13 (19.1)	59 (22.7)	
Cisplatin + Etoposide	8 (11.8)	22 (8.4)	
**Number of days***			
<42	33 (48.5)	114 (43.8)	0.58
≥42	35 (51.5)	146 (56.2)	

RT PTV; The Radiotherapy Planning Target Volume (cm3). **Data available for 180 patients from MSKCC cohort.
*Number of days between end of radiation and durvalumab infusion.

Differences in clinicopathologic characteristics were compared using Pearson's χ^2-test or Fisher's exact test.

Source data are provided as a Source Data file.

Supplementary Table 3. Univariable and multivariable Cox regression analysis.

Progression-free survival	Univariate Hazard Ratio [95%CI]	P-value	Multivariate Hazard ratio [95%CI]	P-value
**Pneumonitis***				
(Discontinued vs Not discontinued)	0.95 [0.63-1.43]	0.79	0.85 [0.51-1.42]	0.55
**ECOG**				
PS 0	Reference	-	Reference	-
PS 1	1.42 [1.03-1.95]	0.03	1.55 [1.03-2.34]	0.03
PS 2	1.76 [0.84-3.69]	0.13	3.37 [1.23-9.21]	0.02
**Stage AJCC 8th**				
IIIA	Reference	-	Reference	-
IIIB	1.62 [1.13-2.31]	0.008	1.48 [0.91-2.41]	0.11
IIIC	2.09 [1.34-3.27]	0.001	2.24 [1.26-4.00]	0.006
**TMB Z-score****	0.66 [0.55-0.78]	2.2e-06	0.63 [0.53-0.76]	2e-06
**PD-L1 TPS**				
<1%	Reference	-	Reference	-
1-49%	1.16 [0.79-1.70]	0.45	1.21 [0.77-1.92]	0.40
50-89%	0.78 [0.50-1.21]	0.26	0.95 [0.59-1.58]	0.92
90-100%	0.36 [0.19-0.69]	0.002	0.43 [0.20-0.93]	0.03
**NLR**				
≥5	Reference		Reference	
<5	0.63 [0.47-0.86]	0.004	0.87 [0.58-1.30]	0.49

Overall survival	Univariate Hazard Ratio [95%CI]	P-value	Multivariate Hazard ratio [95%CI]	P-value
**Pneumonitis***				
(Discontinued vs Not discontinued)	1.14 [0.70-1.87]	0.60	1.34 [0.69-2.60]	0.39
**ECOG**				
PS 0	Reference	-	Reference	-
PS 1	1.44 [0.91-2.26]	0.12	1.39 [0.76-2.54]	0.28
PS 2	2.85 [1.18-6.91]	0.02	3.67 [1.11-12.1]	0.04
**Stage AJCC 8th**				
IIIA	Reference	-	Reference	-
IIIB	1.34 [0.82-2.20]	0.24	1.46 [0.73-2.91]	0.28
IIIC	1.99 [1.09-3.66]	0.03	1.85 [0.82-4.16]	0.13
**TMB Z-score****	0.80 [0.63-1.03]	0.08	0.82 [0.65-1.06]	0.14
**PD-L1 TPS**				
<1%	Reference	-	Reference	-
1-49%	0.80 [0.47-1.35]	0.40	0.61 [0.29-1.23]	0.16
50-89%	0.81 [0.46-1.43]	0.47	1.09 [0.54-2.21]	0.81
90-100%	0.31 [0.12-0.79]	0.01	0.40 [0.13-1.19]	0.09
**NLR**				
≥5	Reference		Reference	
<5	0.61 [0.39-0.94]	0.02	0.76 [0.42-1.37]	0.36

Pneumonitis: time-dependent adjusted. TMB Z-score as continuous variable.
Data are presented as the hazard ratio (HR) with error bars showing 95% confidence interval. Cox proportional hazards models were used to estimate hazard ratios in univariable and multivariable models for progression-free survival (PFS) and overall survival (OS). P-values are according to log-rank test. Source data are provided as a Source Data file.

Supplementary Table 4. Clinicopathologic characteristics of patients who discontinued durvalumab due to pneumonitis (early vs late) and from patients who did not experience pneumonitis.

Clinical Characteristic	Early-onset N=39 (%)	Late-onset N=29 (%)	Not discontinued N=260 (%)	P-value
**Age**				
≥70y	19 (48.7)	16 (55.2)	118 (45.4)	0.58
<70y	20 (51.3)	13 (44.8)	142 (54.6)	
**Sex**				
Male	17 (43.6)	11 (37.9)	142 (54.6)	0.13
Female	22 (56.4)	18 (62.1)	118 (45.4)	
**T Stage**				
0	4 (10.3)	6 (20.7)	38 (14.6)	
1	9 (23.1)	5 (17.2)	49 (18.8)	
2	7 (17.9)	2 (6.9)	46 (17.7)	0.67
3	11 (28.2)	7 (24.1)	51 (19.6)	
4	8 (20.5)	9 (31.0)	76 (29.2)	
**N Stage**				
0	2 (5.1)	3 (10.3)	8 (3.1)	
1	1 (2.6)	2 (6.9)	14 (5.3)	0.55
2	22 (56.4)	15 (51.7)	157 (60.4)	
3	14 (35.9)	9 (31.0)	81 (31.2)	
**Stage (AJCC 8th Edition)**				
IIIA	13 (33.3)	13 (44.8)	95 (36.5)	
IIIB	21 (53.9)	10 (34.5)	127 (48.9)	0.59
IIIC	5 (12.8)	6 (20.7)	38 (14.6)	
**Radiation dose**				
54-58.4 Gy	0	0	12 (4.6)	
60 Gy	35 (89.7)	22 (75.9)	208 (80.0)	0.22
62-70 Gy	4 (10.3)	7 (24.1)	40 (15.4)	
**RT PTV****, median (range)	515 (92-1310)	547 (161-1370)	482 (87-1450)	0.82
**Chemotherapy regimen**				
Carboplatin + Paclitaxel	17 (43.6)	15 (51.7)	119 (45.8)	
Carboplatin + Pemetrexed	11 (28.2)	4 (13.8)	60 (23.1)	0.90
Cisplatin + Pemetrexed	7 (17.9)	6 (20.7)	59 (22.7)	
Cisplatin + Etoposide	4 (10.3)	4 (13.8)	22 (8.4)	
**Number of days***				
<42	19 (48.7)	14 (48.3)	114 (43.8)	0.79
≥42	20 (51.3)	15 (51.7)	146 (56.2)	

RT PTV; The Radiotherapy Planning Target Volume (cm3). **Data available for 180 patients from MSKCC cohort.
*Number of days between end of radiation and durvalumab infusion.

Differences in clinicopathologic characteristics were compared using Pearson’s X2-test or Kruskal-test when appropriate. Source data are provided as a Source Data file.

Supplementary Table 5. PD-L1 antibody clones and dilution of antibodies for immunohistochemistry staining.

Antibody	Clone	Company	Antibody Dilution
**PD-L1**	E1L3N	Cell Signaling	1:300
	22C3	Dako	1:200
	SP263	Ventana	1:200

Supplementary Table 6. Target antigens, antibody clones, and dilution of antibodies for multiplexed immunofluorescence staining.

Antibody	Clone	Company	Antibody Dilution
**CD8**	4B11	Leica	1:200
**PD-L1**	E1L3N	Cell Signaling	1:300
**FOXP3**	D608R	Cell Signaling	1:100
**PD-1**	EPR4877(2)	Abcam	1:300
**Cytokeratin**	AE1/AE3	Agilent	1:100
