# Supplementary material for: Impact of TMB/PD-L1 expression and pneumonitis on chemoradiation and durvalumab response in stage III NSCLC
Source: Nat Commun. 2023 Jul 15;14:4238. doi: 10.1038/s41467-023-39874-8 (PMC10349822; doi:10.1038/s41467-023-39874-8)
Supplement: Supplementary file 3 — Reporting Summary [file 41467_2023_39874_MOESM3_ESM.pdf]

nature portfolio

Corresponding author(s): Narek Shaverdian

Last updated by author(s): 5/12/2023

Reporting Summary

Nature Portfolio wishes to improve the reproducibility of the work that we publish. This form provides structure for consistency and transparency in reporting. For further information on Nature Portfolio policies, see our Editorial Policies and the Editorial Policy Checklist.

Statistics

For all statistical analyses, confirm that the following items are present in the figure legend, table legend, main text, or Methods section.

n/a	Confirmed	
☐	☒	The exact sample size (*n*) for each experimental group/condition, given as a discrete number and unit of measurement
☐	☒	A statement on whether measurements were taken from distinct samples or whether the same sample was measured repeatedly
☐	☒	The statistical test(s) used AND whether they are one- or two-sided<br>*Only common tests should be described solely by name; describe more complex techniques in the Methods section.*
☐	☒	A description of all covariates tested
☐	☒	A description of any assumptions or corrections, such as tests of normality and adjustment for multiple comparisons
☐	☒	A full description of the statistical parameters including central tendency (e.g. means) or other basic estimates (e.g. regression coefficient) AND variation (e.g. standard deviation) or associated estimates of uncertainty (e.g. confidence intervals)
☐	☒	For null hypothesis testing, the test statistic (e.g. *F*, *t*, *r*) with confidence intervals, effect sizes, degrees of freedom and *P* value noted<br>*Give P values as exact values whenever suitable.*
☒	☐	For Bayesian analysis, information on the choice of priors and Markov chain Monte Carlo settings
☒	☐	For hierarchical and complex designs, identification of the appropriate level for tests and full reporting of outcomes
☒	☐	Estimates of effect sizes (e.g. Cohen's *d*, Pearson's *r*), indicating how they were calculated

Our web collection on statistics for biologists contains articles on many of the points above.

Software and code

Policy information about availability of computer code

Data collection	No new codes were generated in this study
Data analysis	RStudio 2022.07.1+554 "Spotted Wakerobin" Release (7872775ebddc40635780ca1ed238934c3345c5de, 2022-07-22) for Windows<br>R v3.6.1 and v3.6.3

For manuscripts utilizing custom algorithms or software that are central to the research but not yet described in published literature, software must be made available to editors and reviewers. We strongly encourage code deposition in a community repository (e.g. GitHub). See the Nature Portfolio guidelines for submitting code & software for further information.

Data

Policy information about availability of data

All manuscripts must include a data availability statement. This statement should provide the following information, where applicable:

- Accession codes, unique identifiers, or web links for publicly available datasets
- A description of any restrictions on data availability
- For clinical datasets or third party data, please ensure that the statement adheres to our policy

Data are available upon reasonable request. The data that support the finding of our study are available upon request from the corresponding author. Sour data for each figure has been provided in this revision. The reason why data is available upon request from the corresponding author is that given the many stake holders involved with this data, having updated accounting of the multiple projects is necessary to avoid duplicative and potentially conflicting conclusions.

Human research participants

Policy information about studies involving human research participants and Sex and Gender in Research.

Reporting on sex and gender	There is no individual level patient data presented.<br>Sex (biological attribute) was taken directly from the clinical chart review.<br>All data was de-identified<br>Sex was used as a covariate in analysis.
Population characteristics	Clinical, pathological and genomic characteristics were abstracted through chart review, Including key variables such as disease stage, age, sex, Eastern Cooperative Oncology Group performance status (ECOG PS), smoking status, tumor histology, neutrophil-to-lymphocyte ratio (NLR) and albumin levels prior durvalumab initiation, PD-L1 expression levels, and TMB.<br><br>PD-L1 expression results were abstracted from clinical data; we did not perform stain for this biomarker; therefore antibodies were not checked as "involved in this study"
Recruitment	Patients were treated as per standard of care. Patients were not recruited for participation.
Ethics oversight	Patients were included if they had consented to each institution's institutional review board-approved medical review protocols. The patient studies were conducted according to the ethical guidelines of the Declaration of Helsinki. De-identified patient data were used from patients who consented to IRB approved protocols Dana-Farber/Harvard Cancer Center 02-180, 11-104,13-364, and/or 17-000 as well as MSK IRB 16-142.

Note that full information on the approval of the study protocol must also be provided in the manuscript.

Field-specific reporting

Please select the one below that is the best fit for your research. If you are not sure, read the appropriate sections before making your selection.

☒ Life sciences ☐ Behavioural & social sciences ☐ Ecological, evolutionary & environmental sciences

For a reference copy of the document with all sections, see nature.com/documents/nr-reporting-summary-flat.pdf

Life sciences study design

All studies must disclose on these points even when the disclosure is negative.

Sample size	No sample size was assessed. This was a retrospective cohort study of all eligible patients treated with standard of care at DFCI and MSKCC between May 2017 to February 2022.
Data exclusions	No data points were excluded.
Replication	Not applicable. Multi-institutional retrospective cohort study of 328 patients treated with standard of care.
Randomization	Not applicable. Our study is a noninterventional retrospective cohort study.
Blinding	Disease status was assessed without knowledge of primary covariates used in analysis.

Reporting for specific materials, systems and methods

We require information from authors about some types of materials, experimental systems and methods used in many studies. Here, indicate whether each material, system or method listed is relevant to your study. If you are not sure if a list item applies to your research, read the appropriate section before selecting a response.

Materials & experimental systems

n/a	Involved in the study	
☒	☐	Antibodies
☒	☐	Eukaryotic cell lines
☒	☐	Palaeontology and archaeology
☒	☐	Animals and other organisms
☐	☒	Clinical data
☒	☐	Dual use research of concern

Methods

n/a	Involved in the study	
☒	☐	ChIP-seq
☒	☐	Flow cytometry
☒	☐	MRI-based neuroimaging

Clinical data

Policy information about clinical studies
All manuscripts should comply with the ICMJEguidelines for publication of clinical research and a completedCONSORT checklist must be included with all submissions.

Clinical trial registration	N/A. Patients treated with standard of care
Study protocol	N/A
Data collection	clinical chart review was used for data collection. Study duration 2017 - 2022.
Outcomes	Physician review and clinical chart review was used to assess for disease status. Chart review was used to obtain data on variables (clinical, genomic, pathological) used in analysis.
